# Supplementary material for: Stable and efficient pure blue quantum-dot LEDs enabled by inserting an anti-oxidation layer
Source: Nat Commun. 2024 Jan 26;15:783. doi: 10.1038/s41467-024-44894-z (PMC10817946; doi:10.1038/s41467-024-44894-z)
Supplement: Supplementary file 1 — Supplementary Information [file 41467_2024_44894_MOESM1_ESM.pdf]

Supplementary Information

Stable and Efficient Pure Blue Quantum-Dot LEDs Enabled by Inserting an Anti-oxidation Layer

Wenjing Zhang[1,†], Bo Li[2,†], Chun Chang[3,†], Fei Chen[1*], Qin Zhang[3], Qingli Lin[1], Lei Wang[1], Jinhang Yan[1], Fangfang Wang[1], Yihua Chong[1], Zuliang Du[1], Fengjia Fan[2*], and Huaibin Shen[1*]

[1] Key Laboratory for Special Functional Materials of Ministry of Education, National & Local Joint Engineering Research Center for High-efficiency Display and Lighting Technology, Henan University, Kaifeng 475004, China

[2] Hefei National Laboratory for Physical Sciences at the Microscale and Department of Modern Physics, CAS Key Laboratory of Microscale Magnetic Resonance, Synergetic Innovation Center of Quantum Information and Quantum Physics, University of Science and Technology of China, Hefei 230026, China

[3] Key Laboratory of Nondestructive Testing Ministry of Education, Nanchang Hangkong University, Nanchang 330063, China

[†]Authors contributed equally.

Correspondence and requests for materials should be addressed to F.C. (email: chenfei.henu@henu.edu.cn), to F.J.F. (email: ffj@ustc.edu.cn) or to H.B.S. (email: shenhuaibin@henu.edu.cn).

Table of contents

1. Supplementary Figures

Supplementary Figures 1-12

2. Supplementary Table

Supplementary Table 1

3. Supplementary Method

Supplementary Method 1

4. Supplementary References

Supplementary References 1-41

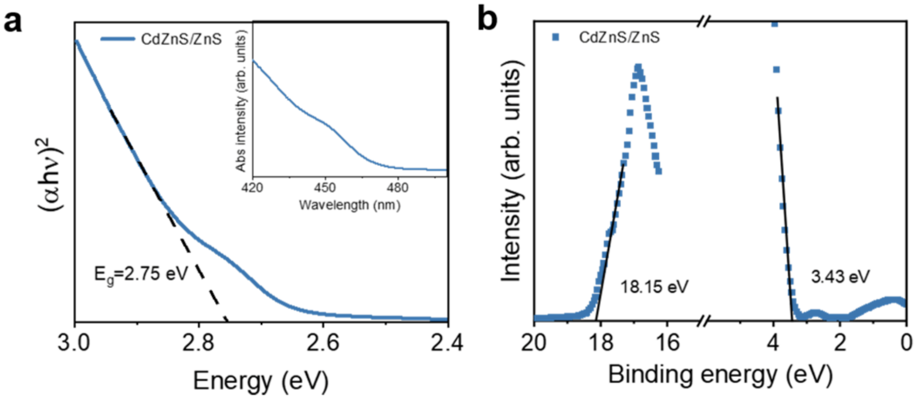

Supplementary Fig. 1 | Ultraviolet photoelectron spectroscopy (UPS) measurement of CdZnS/ZnS core/shell quantum-dots (QDs). a, The Tauc plot of QDs between $(\alpha h\nu)^2$ and photon energy. The inset is the absorption of QDs. **b,** UPS spectra of the high-binding energy secondary electron cutoff regions and the valence-band edge regions of QDs.

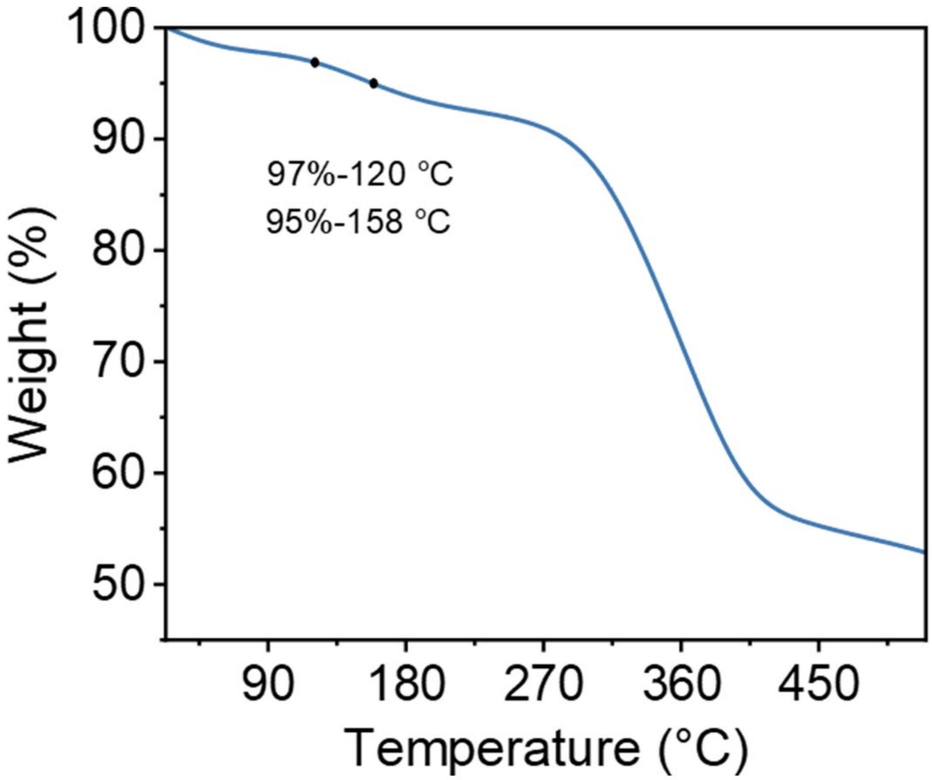

Supplementary Fig. 2 | Thermogravimetric (TGA) measurement. TGA data analysis of PBO materials.

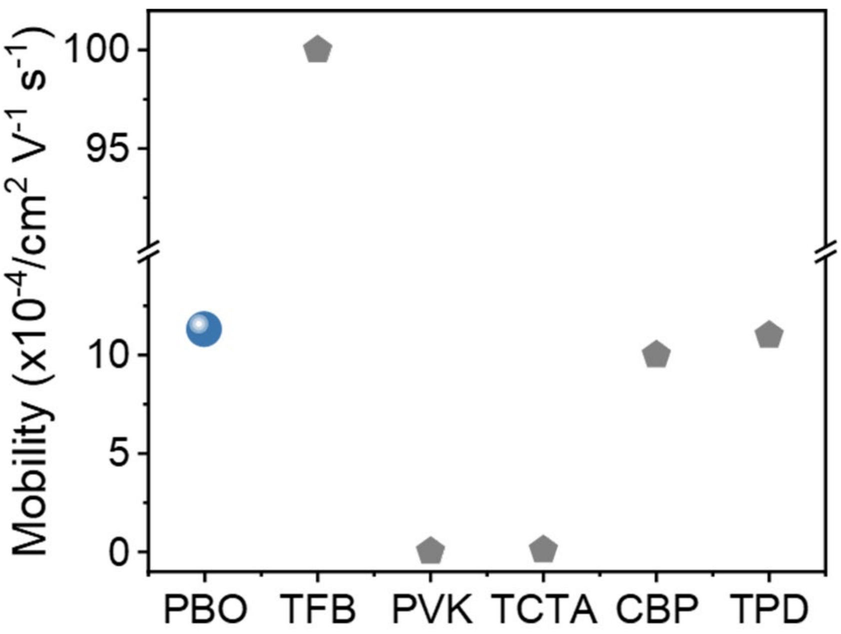

Supplementary Fig. 3 | Hall effect measurements. The hole mobility of PBO, TFB, PVK, TCTA, CBP and TPD.

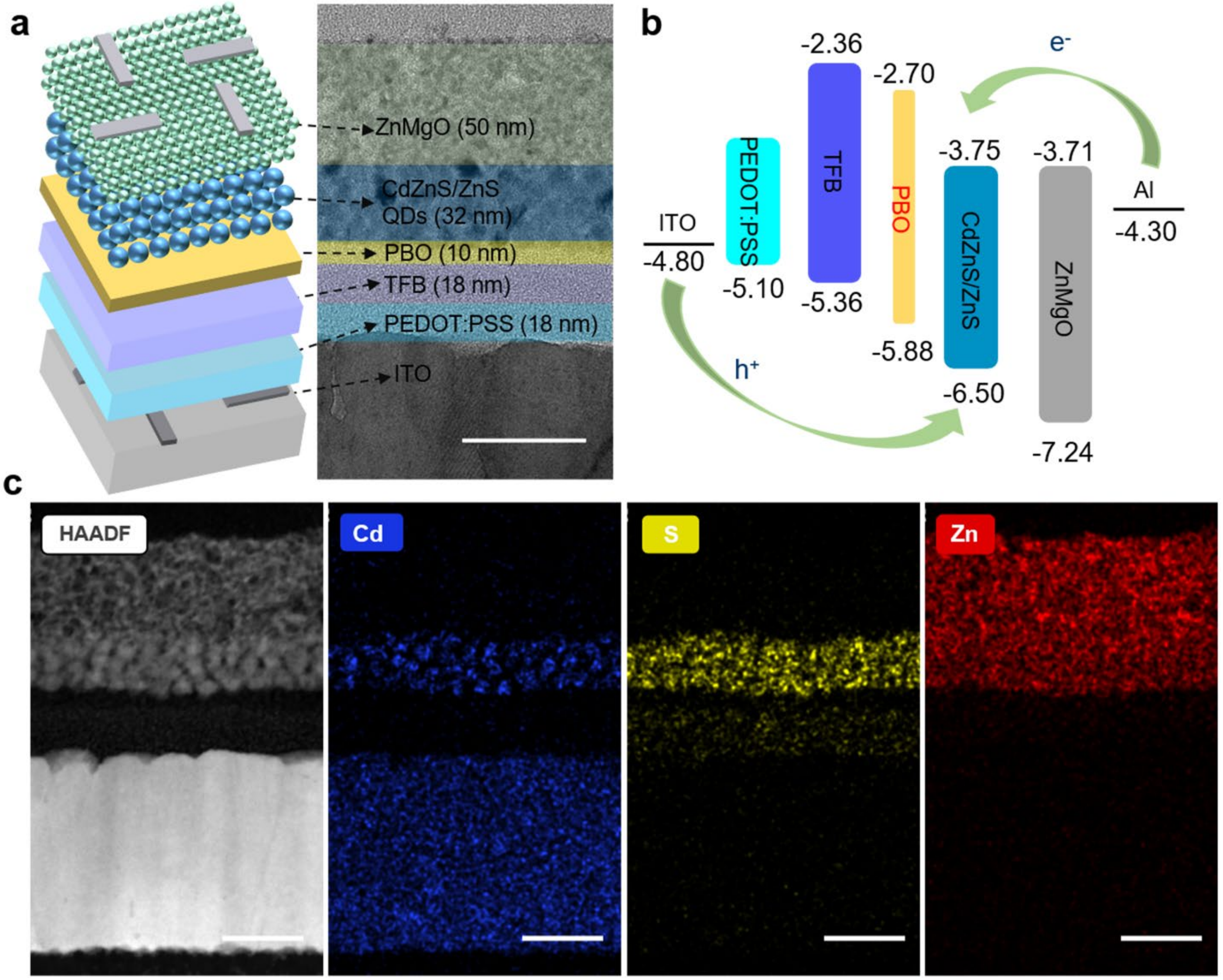

Supplementary Fig. 4 | Quantum-dot light-emitting diode (QD-LED) structure and characterizations. **a,** Schematic device structure, cross-sectional scanning electron microscope (SEM) image of QD-LED. Scale bar: 50 nm. **b,** The energy level diagram of QD-LED. **c,** Element distribution of each layer. Scale bar:50 nm.

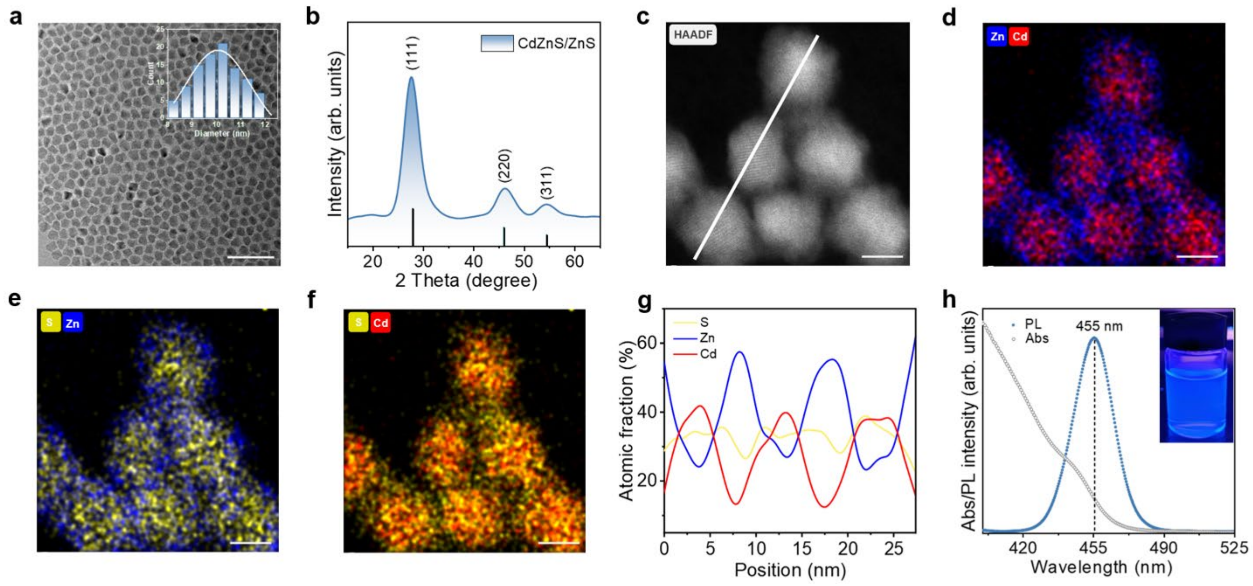

Supplementary Fig. 5 | QDs characterizations. a, Transmission electron microscopy (TEM) image and histogram of diameters of 100 CdZnS/ZnS QDs. Scale bar: 50 nm. **b,** X-ray diffraction (XRD) pattern of as-prepared QDs. The black line indicates the peak positions of standard references of bulk zinc-blende ZnS. **c,** A high-angle annular dark field (HAADF) image. Scale bar: 5 nm. **d-f,** Energy dispersive spectroscopy (EDS) elemental mapping of Zn, Cd and S elements from several CdZnS/ZnS core/shell QDs shown in c. Scale bar: 5 nm. **g,** Elements line scan spectra in c. **h,** Absorption and photoluminescence (PL) spectra of CdZnS/ZnS core/shell QDs. The inset is the photograph of QD-octane dispersion under ultraviolet (UV) irradiation.

According to the TEM image, and XRD pattern in Supplementary Fig. 4a-b, the core/shell QDs have an average diameter of about 10 nm with zincblende crystal structure. From the EDS elemental mapping (Supplementary Fig. 4d-g), the Cd atoms mainly locate at the QD cores, and the S atoms are distributed throughout the core/shell regions. The Zn atoms exist in the core but are mainly distributed in the shell, corresponding to the element line scan spectra. The absorption, PL spectra, and photograph of QDs under

UV irradiation are shown in Supplementary Fig. 4h, they show pure blue color.

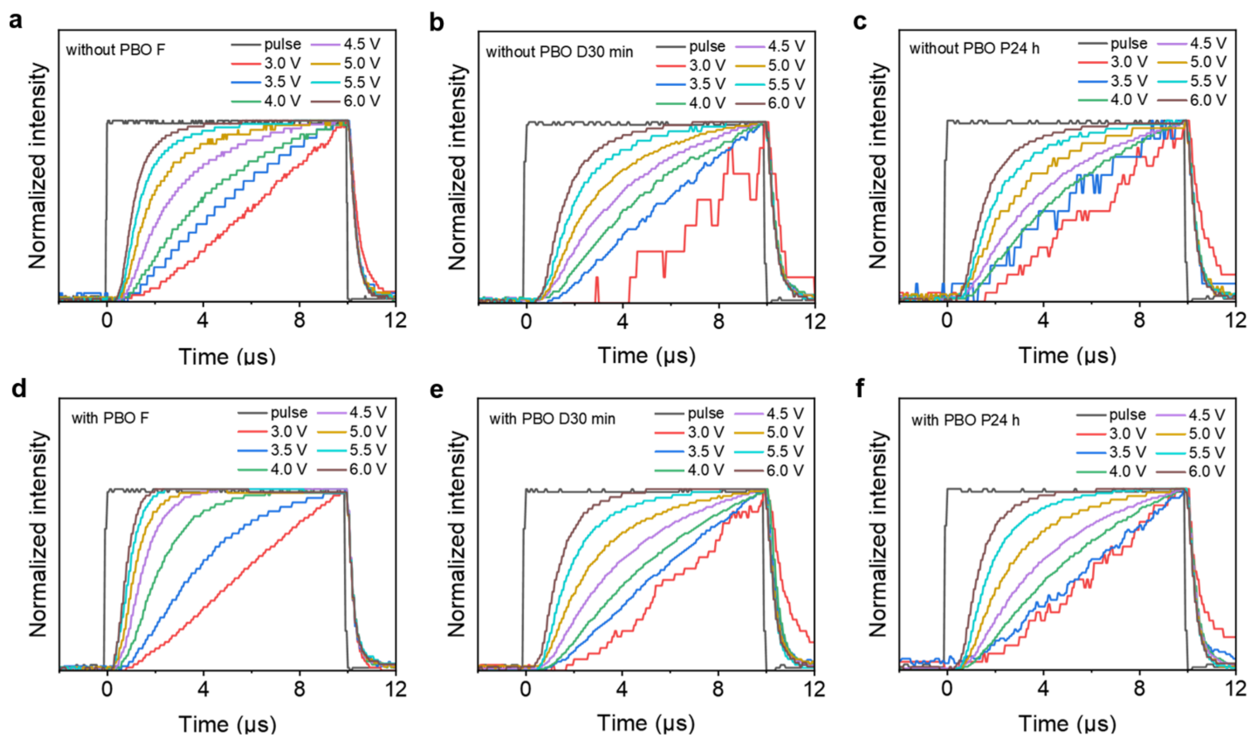

Supplementary Fig. 6 | Transient electroluminescence (tr-EL) measurements. Tr-EL spectra for the devices without PBO **a,** fresh (F), **b,** after degradation (turn on for 30 min) (D30 min) and **c,** 24 h after degradation (P24 h). Tr-EL spectra for the devices with PBO **d,** fresh, **e,** after degradation (turn on for 30 min) and **f,** 24 h after degradation.

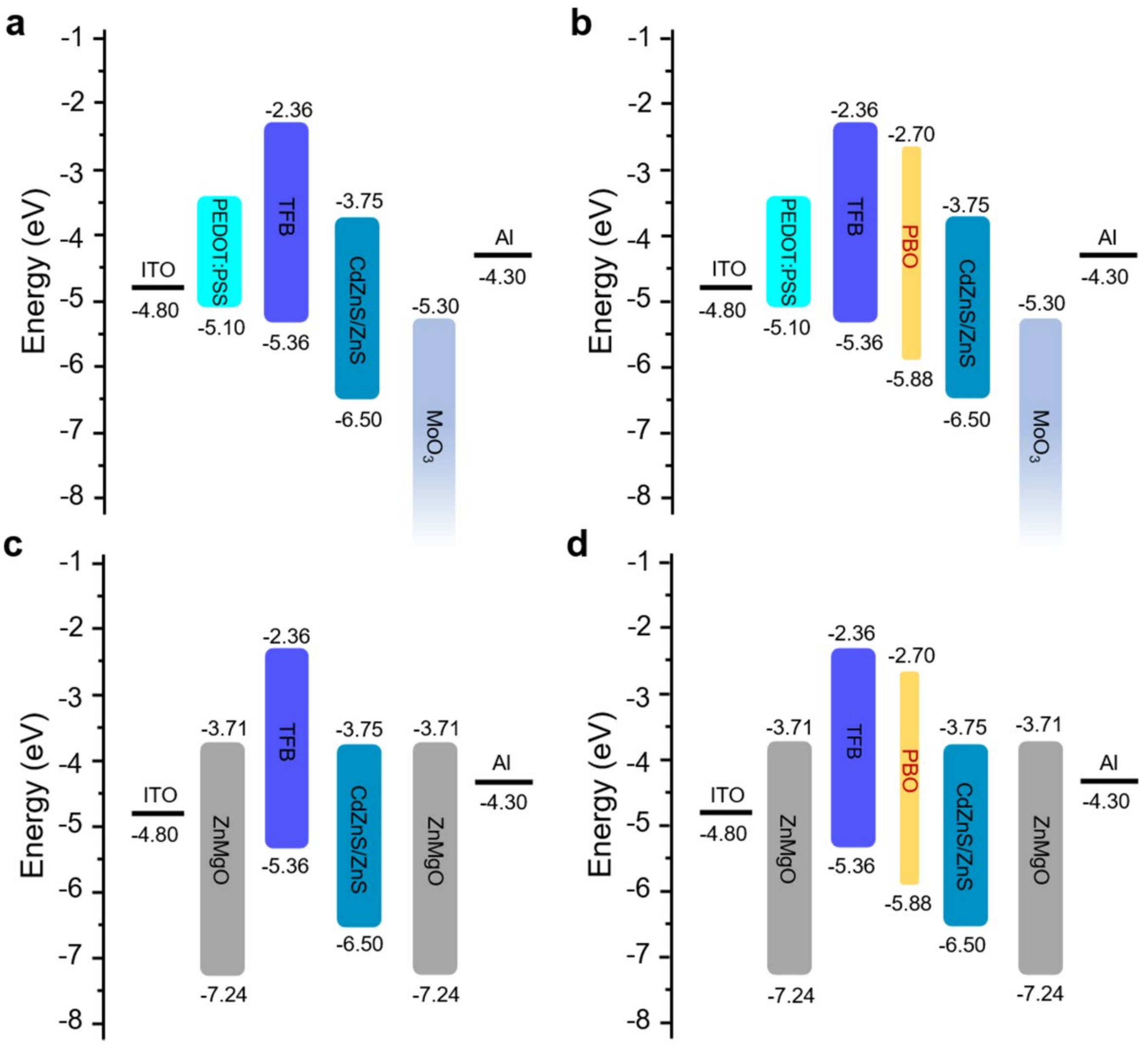

Supplementary Fig. 7 | Energy level diagrams of Hole-only-device (HOD) and Electron-only-device (EOD) devices. HOD with **a,** ITO/PEDOT:PSS/TFB/QDs/MoO_3/Al and **b,** ITO/PEDOT:PSS/TFB/PBO/QDs/MoO_3/Al. EOD with **c,** ITO/ZnMgO/TFB/QDs/ZnMgO/Al and **d,** ITO/ZnMgO/TFB/PBO/QDs/ZnMgO/Al.

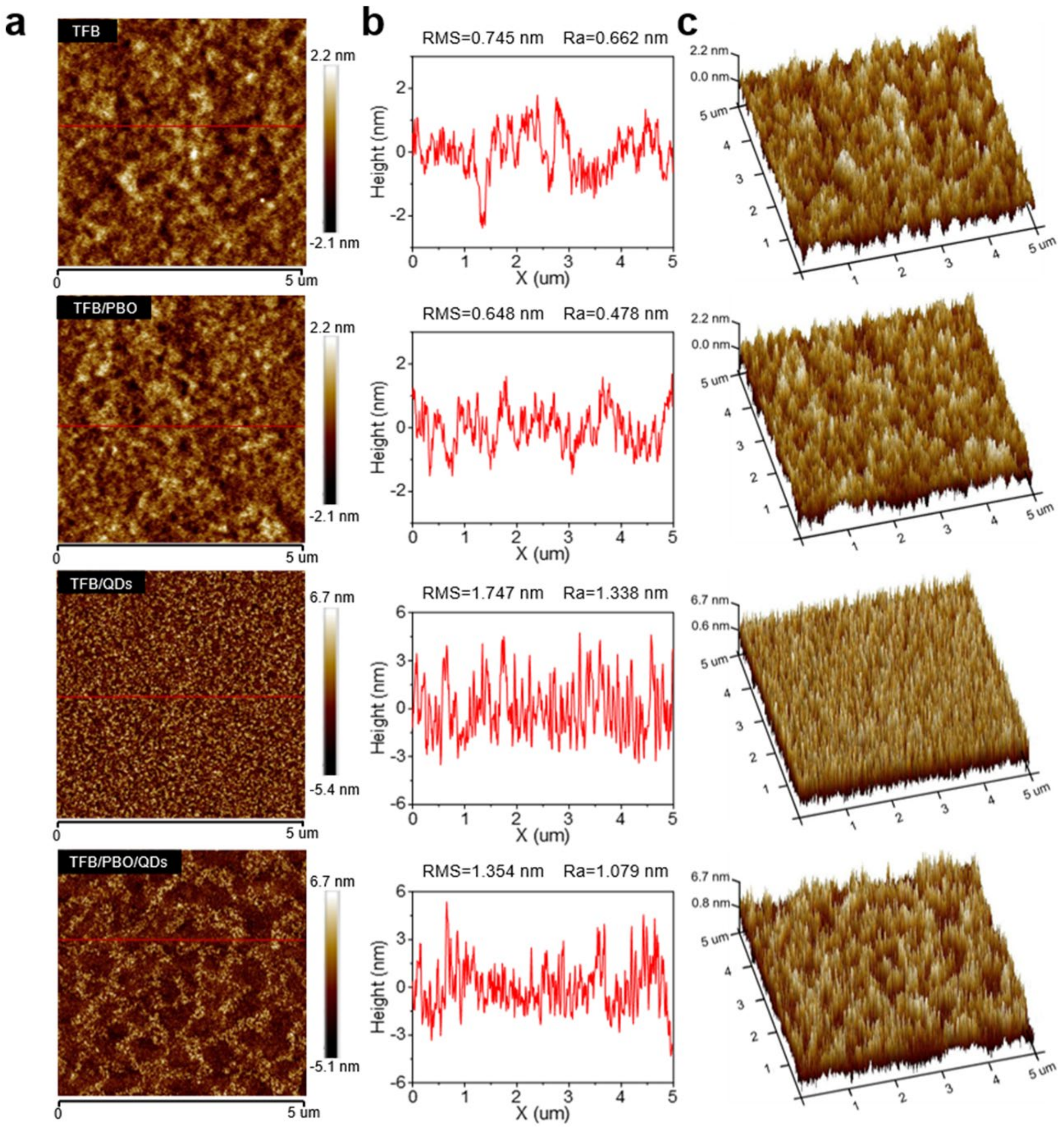

Supplementary Fig. 8 | Atomic force microscope (AFM) measurements of TFB, TFB/PBO, TFB/QD, TFB/PBO/QD films. a, AFM topographies, **b,** line scan and **c,** 3D images.

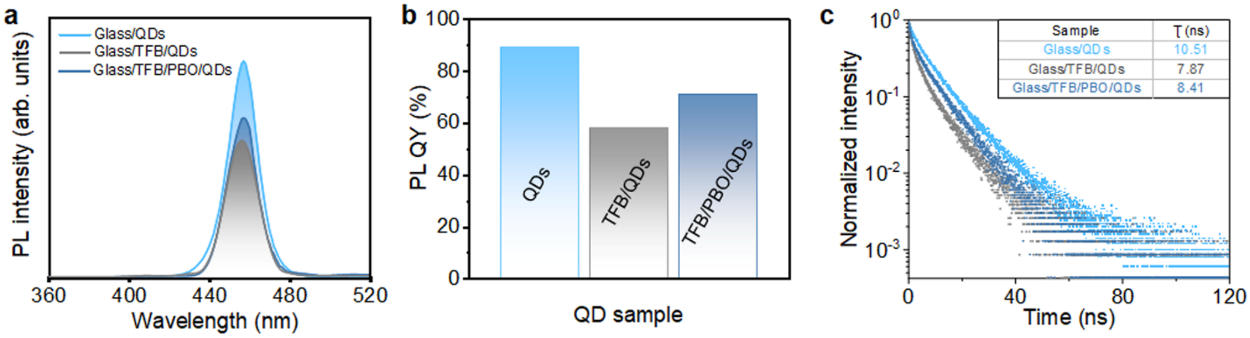

Supplementary Fig. 9 | QDs film characterizations. a, PL spectra, **b,** Photoluminescence quantum yield (PL QY) data, and **c,** PL decay curves of QD, TFB/QD, TFB/PBO/QD film on glass substrate.

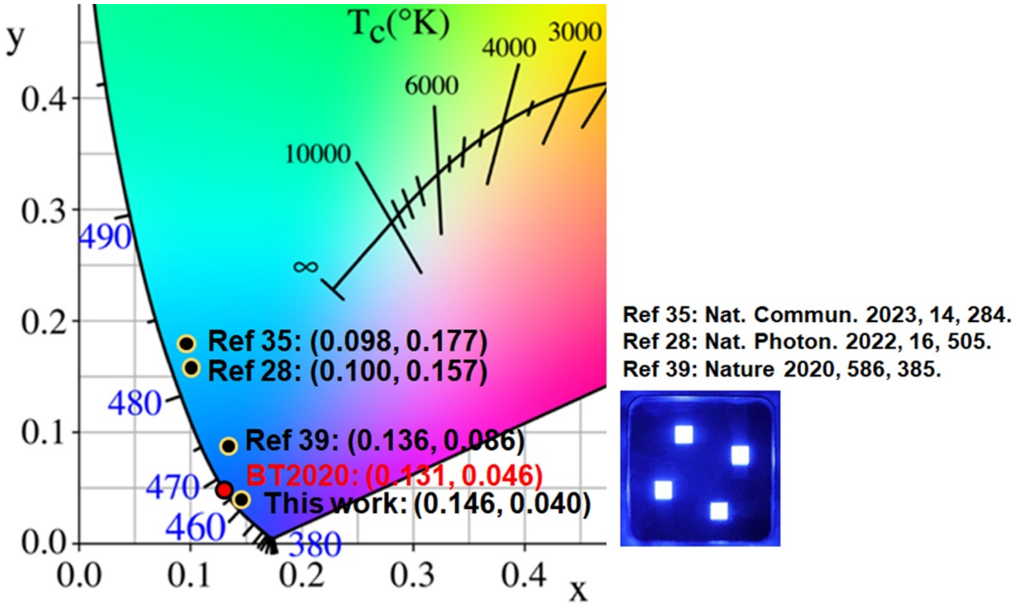

Supplementary Fig. 10 | Commission Internationale de l'Eclairage (CIE) chromaticity coordinates.

CIE chromatic coordinates of references, BT2020 and our QD-LED.

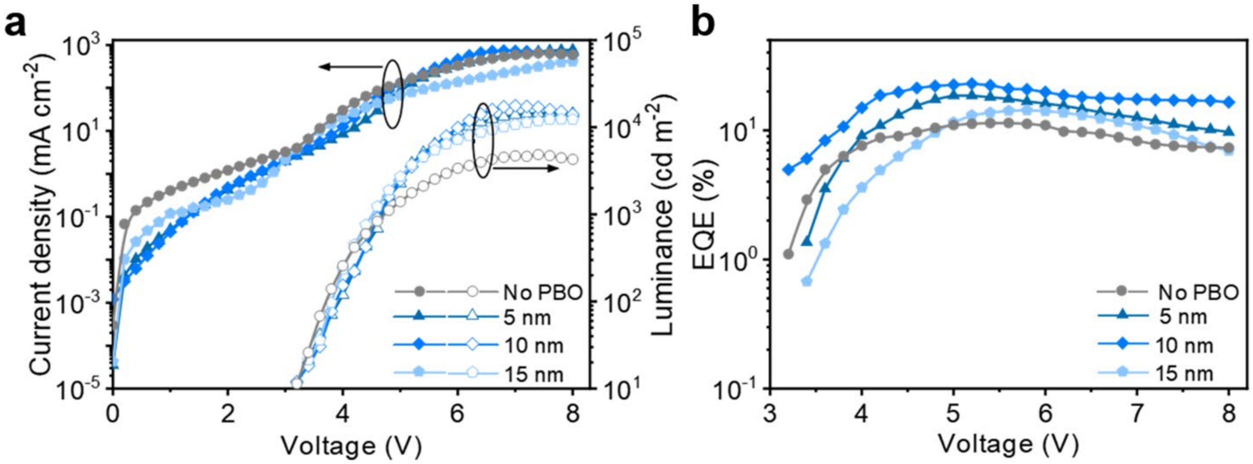

Supplementary Fig. 11 | Device performances of QD-LEDs with PBO layers of different thicknesses.

a, The current density-luminance-voltage characteristics and **b,** EQE as a function of voltage curves.

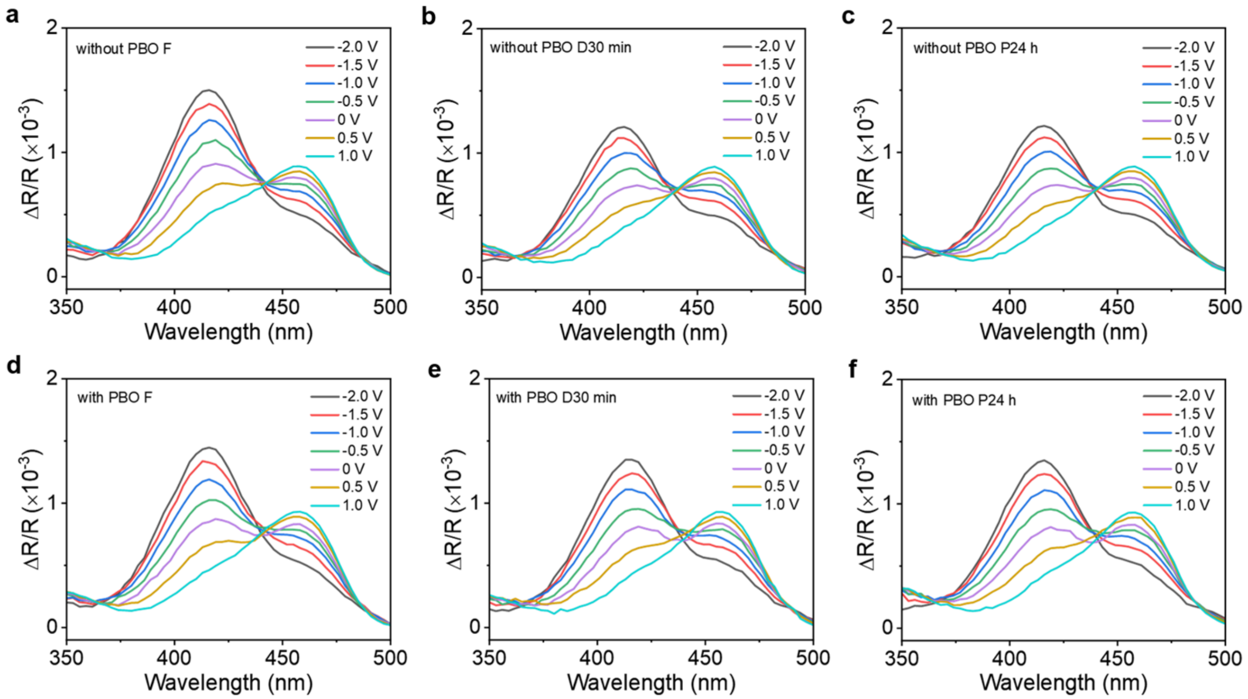

Supplementary Fig. 12 | Electro-absorption (EA) measurements for the devices without and with PBO. The EA spectra for the devices without PBO **a,** fresh, **b,** after degradation (turn on for 30 min) and **c,** 24 h after degradation. The EA spectra for the devices with PBO **d,** fresh, **e,** after degradation (turn on for 30 min) and **f,** 24 h after degradation.

Supplementary Table. 1 | Statistics of blue QD-LEDs performance. Comparison of state-of-the-art blue QD-LEDs in previous literature with the current work.

Blue QD-LEDs	Year	Structure	EL (nm)	EQE (%)	L_{max} (cd m^{-2})	T_{50} (h)	CIE	Ref.
Deep blue	2012	ITO/PEDOT:PSS/PVK/ZnSe/ZnS/ZnO/Al	430	0.65	--	--	--	1
	2012	ITO/ZnO/CdZnS@ZnS/CBP/MoO_3/Al	437	1.7	2250	--	(0.170, 0.020)	2
	2013	ITO/PEDOT:PSS/PVK/ZnCdS/ZnS/ ZnO/Al	452	7.1	2624	--	(0.153, 0.027)	3
	2014	ITO/PEDOT:PSS/TFB/$Zn_xCd_{1-x}S$/ZnS/ ZnO/Al	445	3.8	4100	--	--	4
	2015	ITO/PEDOT:PSS/TFB/ZnCdS/ZnS-OT/ ZnO/Al	443	12.2	7600	--	(0.140, 0.020)	5
	2015	ITO/PEDOT:PSS/PVK/ZnSe/ZnS/ZnO NPs/Al	429	7.83	2623	--	(0.169, 0.023)	6
	2015	ITO/PEDOT:PSS/TFB:PVK/ZnSe/ZnS/ZnO NPs/Al	430	7.39	2856	--	--	7
	2018	ITO/PEDOT:PSS/PVK/ZnCdS/10$Cd_xZn_{1-x}S$/2ZnS/ZnO NPs/Al	445	18.0	6768	47.4	--	8
	2019	ITO/PEDOT:PSS/TFB/ZnCdS/ZnS/TBS-PBO/ZnO:Mg/Al	452	17.4	7992	--	(0.150, 0.030)	9
	2019	ITO/PEDOT:PSS/PVK/ZnSeTe/ZnSe/ZnS/ZnMgO/Al	445	4.2	1195	14	--	10
	2020	ITO/PEDOT:PSS/PVK/ZnSeTe/ZnSe/ZnSeS/ZnS/ZnMgO-$Mg(OH)_2$/Al	447	9.5	2904	--	(0.148, 0.048)	11
	2020	ITO/PEDOT:PSS/PVK/ZnSe/ZnS/ZnMgO/Al	428	14.7	390	--	(0.169, 0.015)	12
			445	10.7	--	--	(0.157, 0.021)	
	2021	ITO/PEDOT:PSS/PVK/ZnSe/ZnS/ZnMgO/Al	445	12.2	1055	237	(0.160, 0.030)	13
	2022	ITO/PEDOT:PSS/PVK/ZnSe/ZnS/ZnMgO/Al	443	13.6	1031	305	(0.170, 0.030)	14
Sky blue	2011	ITO/PEDOT:PSS/Poly-TPD/CdSe/ZnS/ZnO/Al	470	0.22	4200	--	--	15
	2017	ITO/PEDOT:PSS/PVK/CdSe/ZnS/ZnO NPs/Al	468	19.8	4890	47.5	(0.136, 0.078)	16
	2017	ITO/ZMO/InP/ZnS//ZnS/CBP/MoO_3/Al	488	--	90	--	--	17
	2018	ITO/PEDOT:PSS/TFB/ZnCdSe/ZnS//ZnS/PMMA/ZnO/Al	479	16.2	14100	355	(0.119, 0.154)	18
	2019	ITO/PEDOT:PSS/TFB/CdSe/ZnSe/ZnO/Al	480	8.05	62600	7000	--	19
	2019	ITO/NiO/TFB/PVK/$CsFAPbBr_3$/TPBi/LiF/Al	483	9.5	700	250 s	--	20
	2020	ITO/PEDOT:PSS/TFB/ZnCdSeS/ZnO/Al	480	9.9	52360	2630	--	21
	2020	ITO/PEDOT:PSS/Poly-TPD/PVK/CdSeS/ZnSeS/ZnS/ZnMgO/Al	480	10.0	--	10000	--	22
	2020	ITO/PEDOT:PSS/TFB/InP/GaP/ZnS//ZnS/ZnO/Al	488	1.01	3120	2	--	23
	2020	ITO/PEDOT:PSS/PVK/InGaP/ZnSeS/ZnS/ZnMgO/Al	469	2.5	1038	--	--	24
	2021	ITO/PEDOT:PSS/TFB /InP/ZnS/ZnS/ZnO/Al	484	1.47	125	--	--	25
	2021	ITO/PEDOT:PSS/$CsPbBr_{3-x}Cl_x$/TPBi/LiF/Al	486	16.8	1694	--	(0.083, 0.221)	26
	2021	ITO/PEDOT:PSS/PVK/(Cs/Rb/K/PEA)Pb$(Br/Cl)_3$/DPEPO/TPBi/LiF/Al	488	10.17	6728	--	--	27
	2022	ITO/PEDOT:PSS/PF8Cz/CdZnSe/ZnS/ZnMgO/Al	479	21.9	--	24000	(0.100, 0.157)	28
	2022	ITO/PEDOT:PSS/PVK/ZnSe/ZnS/ZnCdSe/ZnS/ZnMgO/Al	475	20.6	24020	2297	--	29
	2022	ITO/PEDOT:PSS/TFB / InP_{ESC}@ZnS/ZnMgO/Al	487	1.4	1162	--	--	30
	2022	ITO/PEDOT:PSS/TFB /InP/ZnS/ZnS/ZnMgO/Al	488	2.6	422	--	--	31
	2022	ITO/PEDOT:PSS/PVK/$CsPbBr_3$/D-ZnO/Ag	470	8.7	11100	15	(0.130, 0.060)	32

	2022	ITO/PEDOT:PSS/Poly-TPD/$CsPb(Br_xCl_{1-x})_3$ /TPBi/LiF/Al	490	14.6	403	12min	(0.090, 0.260)	33
	2023	ITO/PEDOT:PSS/PTAA/ $FA_{1-x}Cs_xPbBr_{1-x}Cl_x$/ETM-$B_2$/LiF /Al	490	13.17	8656	20min	--	34
	2023	ITO/PEDOT:PSS/TFB/ZnCdSe/ZnCdSeS/ZnS/ZnO/Al	482	20.4	--	80377	(0.098, 0.177)	35
Pure blue	2007	ITO/PEDOT:PSS/Poly-TPD/CdS/ZnS/Al	460	--	1600	--	--	36
	2015	ITO/PEDOT:PSS/PVK/$Cd_{1-x}Zn_xS$@ZnS/ ZnO/Al	455	10.7	4000	1000	(0.160, 0.020)	37
	2017	ITO/PEDOT:PSS/TFB/$Zn_xCd_{1-x}S$/ZnS/ZnO NPs/Al	455	11.9	10400	4300	--	38
		ITO/PEDOT:PSS/PVK/$Zn_xCd_{1-x}S$/ZnS/ZnO NPs/Al		15.6	4500	47		
	2020	ITO/PEDOT:PSS/TFB/ZnTeSe/ZnSe/ZnS-Cl(f)/QD(l)/ZnMgO/Al	460	20.2	88900	15850	(0.136, 0.086)	39
	2021	ITO/PEDOT:PSS/CNPr-TFB/QD/ZnMgO/Al	463	11.3	≈8000	15.8	(0.130, 0.070)	40
	2022	ITO/PEDOT:PSS/CBP-V:T5DP-2,7/QDs/ZnO:PVP/Al	461	18.59	44080	502	(0.140, 0.040)	41
	2023	ITO/PEDOT:PSS/TFB/PBO/CdZnS/ZnS/ZnMgO/Al	458	23.0	17641	41022	(0.146, 0.040)	This work

Supplementary Method 1

Fabrication of electron-only-device and hole-only-device

The structure of EODs is: ITO/ZnMgO/TFB/QDs/ZnMgO/Al and ITO/ZnMgO/TFB/PBO/QDs/ZnMgO/Al. HODs were fabricated with a structure of ITO/PEDOT:PSS/TFB/QDs/MoO_3/Al and ITO/PEDOT:PSS/TFB/PBO/QDs/MoO_3/Al. The EODs and HODs were fabricated using ITO glass substrates. Before use, these substrates were ultrasonically cleaned with detergent, deionized water, acetone, and isopropanol for 15 min, respectively, and then treated with UV ozone for 15 min.

For fabricating the EODs, ZnMgO (20 mg mL^{-1}) was spin-coated at 2000 rpm for 30 s, followed by baking at 80 °C for 30 min. Then, TFB was spin-coated at a concentration of 6 mg mL^{-1} in chlorobenzene (3000 rpm for 50 s), and then baked at 150 °C for 30 min. Next, the PBO solution in DMF was spin-coated at 3000 rpm for 30 s and baked at 120 °C for 30 min. Subsequently, the blue CdZnS/ZnS QDs solution in n-octane was then spin-coated at 2000 rpm for 40 s. In turn, ZnMgO (20 mg mL^{-1}) was spin-coated at 2000 rpm for 30 s, followed by baking at 80 °C for 30 min. Finally, an Al anode was deposited via thermal evaporation at a rate of ≈ 0.1 nm s^{-1} under a high vacuum of 4×10^{-6} Torr.

For fabricating the HODs, the mixed solution (1:1) of PEDOT:PSS (poly(3,4-ethylenedioxythiophene)/poly(styrenesulfonate), AI 4083) and isopropanol used as HILs, was spin-coated onto the ITO substrates at the spin speed of 5500 rpm and baked at 150 °C for 15 min in air. Then, these substrates were immediately transferred to the N_2-filled glove box for spin-coating of TFB, PBO and QDs. TFB was spin-coated at a concentration of 6 mg mL^{-1} in chlorobenzene (3000 rpm for 50 s), and then baked

at 150 °C for 30 min. Next, the PBO solution in DMF was spin-coated at 3000 rpm for 30 s and baked at 120 °C for 30 min. Subsequently, the blue CdZnS/ZnS QDs solution in n-octane was then spin-coated at 2000 rpm for 40 s. In turn, MoO_3 and Al anode were deposited via thermal evaporation at a rate of ≈ 0.1 nm s^{-1} under a high vacuum of 4×10^{-6} Torr, forming a 10 nm MoO_3 layer and 100 nm anode.

Supplementary References

1. Xiang, C. et al. Solution processed multilayer cadmium-free blue/violet emitting quantum dots light emitting diodes. *Appl. Phys. Lett*. **101**, 053303 (2012).

2. Kwak, J. et al. Bright and efficient full-color colloidal quantum dot light-emitting diodes using an inverted device structure. *Nano Lett*. **12**, 2362-2366 (2012).

3. Lee, K. H. et al. Highly efficient, color-pure, color-stable blue quantum dot light-emitting devices. *ACS Nano* **7**, 7295-7302 (2013).

4. Shen, H. et al. High-efficient deep-blue light-emitting diodes by using high quality $Zn_xCd_{1-x}S$/ZnS core/shell quantum dots. *Adv. Funct. Mater.* **24**, 2367-2373 (2014).

5. Shen, H. et al. High-efficiency, low turn-on voltage blue-violet quantum-dot-based light-emitting diodes. *Nano Lett*. **15**, 1211-1216 (2015).

6. Wang, A. et al. Bright, efficient, and color-stable violet ZnSe-based quantum dot light-emitting diodes. *Nanoscale* **7**, 2951-2959 (2015).

7. Lin, Q. et al. Cadmium-free quantum dots based violet light-emitting diodes: High-efficiency and brightness via optimization of organic hole transport layers. *Org. Electron*. **25**, 178-183 (2015).

8. Wang, O. et al. High-efficiency, deep blue ZnCdS/$Cd_xZn_{1-x}S$/ZnS quantum-dot-light-emitting devices with an EQE exceeding 18%. *Nanoscale* **10**, 5650-5657 (2018).

9. Li, D. et al. Blue quantum dot light-emitting diodes with high luminance by improving the

charge transfer balance. *Chem. Commun*. **55**, 3501-3504 (2019).

10. Jang, E. P. et al. Synthesis of alloyed ZnSeTe quantum dots as bright, color-pure blue emitters. *ACS Appl. Mater. Inter*faces **11**, 46062-46069 (2019).

11. Han, C. -Y. et al. More than 9% efficient ZnSeTe quantum dot-based blue electroluminescent devices. *ACS Energy Lett*. **5**, 1568-1576 (2020).

12. Ryowa, T. et al. High-efficiency quantum dot light-emitting diodes with blue cadmium-free quantum dots. *J. Soc. Inf. Display* **28**, 401-409 (2020).

13. Gao, M. et al. Bulk-like ZnSe quantum dots enabling efficient ultranarrow blue light-emitting diodes. *Nano Lett*. **21**, 7252-7260 (2021).

14. Gao, M. et al. Alleviating electron over-injection for efficient cadmium-free quantum dot light-emitting diodes toward deep-blue emission. *ACS Photonics* **9**, 1400-1408 (2022).

15. Qian, L. et al. Stable and efficient quantum-dot light-emitting diodes based on solution-processed multilayer structures. *Nat. Photonics* **5**, 543-548 (2011).

16. Wang, L. et al. Blue quantum dot light-emitting diodes with high electroluminescent efficiency. *ACS Appl. Mater. Inter*faces **9**, 38755-38760 (2017).

17. Shen, W. et al. Synthesis of highly fluorescent InP/ZnS small-core/thick-shell tetrahedral-shaped quantum dots for blue light-emitting diodes. *J. Mater. Chem. C* **5**, 8243-8249 (2017).

18. Lin, Q. et al. Nonblinking quantum-dot-based blue light-emitting diodes with high efficiency and a balanced charge-injection process. *ACS Photonics* **5**, 939-946 (2018).

19. Shen, H. et al. Visible quantum dot light-emitting diodes with simultaneous high brightness and efficiency. *Nat. Photonics* **13**, 192-197 (2019).

20. Liu, Y. et al. Efficient blue light-emitting diodes based on quantum-confined bromide perovskite nanostructures. *Nat. Photonics* **13**, 760-764 (2019).

21. Wang, F. et al. Suppressed efficiency roll-off in blue light-emitting diodes by balancing the spatial charge distribution. *J. Mater. Chem. C* **8**, 12927-12934 (2020).

22. Pu, C. et al. Electrochemically-stable ligands bridge the photoluminescence-electroluminescence gap of quantum dots. *Nat. Commun*. **11**, 937 (2020).

23. Zhang, H. et al. High-brightness blue InP quantum dot-based electroluminescent devices: the role of shell thickness. *J. Phys. Chem. Lett*. **11**, 960-967 (2020).

24. Kim, K. -H. et al. Cation-exchange-derived InGaP alloy quantum dots toward blue emissivity. *Chem. Mater*. **32**, 3537-3544 (2020).

25. Yu, P. et al. Inorganic solid phosphorus precursor of sodium phosphaethynolate for synthesis of highly luminescent InP-based quantum dots. *ACS Energy Lett*. **6**, 2697-2703 (2021).

26. Shen, Y. et al. Interfacial nucleation seeding for electroluminescent manipulation in blue perovskite light-emitting diodes. *Adv. Funct. Mater*. **31**, 2103870 (2021).

27. Zhu, C. et al. High triplet energy level molecule enables highly efficient sky-blue perovskite light-emitting diodes. *J. Phys. Chem. Lett*. **12**, 11723-11729 (2021).

28. Deng, Y. Z. et al. Solution-processed green and blue quantum-dot light-emitting diodes with

eliminated charge leakage. *Nat. Photonics* **16**, 505-511 (2022).

29. Wang, F. et al. High-performance blue quantum-dot light-emitting diodes by alleviating electron trapping. *Adv. Opt. Mater*. **10**, 2200319 (2022).

30. Suh, Y. H. et al. Engineering core size of InP quantum dot with incipient ZnS for blue emission. *Adv. Opt. Mater*. **10**, 2102372 (2022).

31. Zhang, W. et al. High quantum yield blue InP/ZnS/ZnS quantum dots based on bromine passivation for efficient blue light-emitting diodes. *Adv. Opt. Mater*. **10**, 2200685 (2022).

32. Liu, A., Bi, C. & Tian, J. All solution-processed high performance pure-blue perovskite quantum-dot light-emitting diodes. *Adv. Funct. Mater.* **32**, 2207069 (2022).

33. Zhu, H. et al. Enriched-bromine surface state for stable sky-blue spectrum perovskite QLEDs with an EQE of 14.6. *Adv. Mater*. **34**, 2205092 (2022).

34. Yuan, S. et al. Balancing charge injection via a tailor-made electron-transporting material for high performance blue perovskite QLEDs. *ACS Energy Lett*. **8**, 818-826 (2023).

35. Chen, X. et al. Blue light-emitting diodes based on colloidal quantum dots with reduced surface-bulk coupling. *Nat. Commun.* **14**, 284 (2023).

36. Tan, Z. et al. Bright and color-saturated emission from blue light-emitting diodes based on solution-processed colloidal nanocrystal quantum dots. *Nano Lett*. **7**, 3803-3807 (2007).

37. Yang, Y. et al. High-efficiency light-emitting devices based on quantum dots with tailored nanostructures. *Nat. Photonics* **9**, 259-266 (2015).

38. Shen, H. et al. Efficient and long-lifetime full-color light-emitting diodes using high luminescence quantum yield thick-shell quantum dots. *Nanoscale* **9**, 13583-13591 (2017).

39. Kim, T. et al. Efficient and stable blue quantum dot light-emitting diode. *Nature* **586**, 385-389 (2020).

40. Wu, W. et al. An efficient hole transporting polymer for quantum dot light-emitting diodes. *Adv. Mater. Interfaces* **8**, 2100731 (2021).

41. Zhang, X. et al. Constructing effective hole transport channels in cross-linked hole transport layer by stacking discotic molecules for high performance deep blue QLEDs. *Adv. Sci.* **9**, 2200450 (2022).
